# Effects of floral symmetry and orientation on the consistency of pollinator entry angle

**DOI:** 10.1007/s00114-023-01845-w

**Published:** 2023-05-16

**Authors:** Nina Jirgal, Kazuharu Ohashi

**Affiliations:** 1 University of Manchester, Oxford Road, Manchester, M13 9PL UK; 2 Graduate School of Life and Environmental Sciences, University of Tsukuba, Tsukuba, Ibaraki 305-8572 Japan

**Keywords:** Approach consistency, Artificial flowers, *Bombus*, Floral orientation, Floral symmetry, Pollen placement

## Abstract

Since the publication of Sprengel’s (1793) observations, it has been considered that flowers with zygomorphic (or bilaterally symmetrical) corollas evolved to restrict the movement of pollinators into the flower by limiting the pollinator’s direction of approach. However, little empirical support has been accumulated so far. Our aim was to build on previous research that showed zygomorphy reduces variance in pollinator entry angle, aiming to observe whether floral symmetry or orientation had an impact on pollinator entry angle in a laboratory experiment using bumble bees, *Bombus ignitus*. Using nine different combinations of artificial flowers created from three symmetry types (radial, bilateral and disymmetrical) and three orientation types (upward, horizontal, and downward), we tested the effects of these two floral aspects on the consistency of bee entry angle. Our results show that horizontal orientation significantly reduced the variance in entry angle, while symmetry had little effect. We also found either little or no significant interactions between angle and symmetry in their effect on entry angle. Thus, our results suggest that horizontal orientation forces the bees to orient themselves relative to gravity rather than the corolla and stabilizes their flower entry. This stabilizing effect may have been mistaken for the effect of zygomorphic corolla as it is presented horizontally in most species. Consequently, we suggest that the evolution of horizontal orientation preceded that of zygomorphy as indicated by some authors, and that the reason behind the evolution of zygomorphy should be revisited.

**Supplementary Information:**

The online version contains supplementary material available at 10.1007/s00114-023-01845-w.

## Introduction

Zygomorphy, or bilateral symmetry, in angiosperm flowers is suggested to have evolved independently in multiple lineages from their ancestral radial form about 50MY after their initial emergence, which coincides with the emergence of specialised pollinators (Citerne et al. 2010; Hileman 2014). Currently, 130 origins of zygomorphy have been estimated, while only 69 reversions to actinomorphy, or radial symmetry, have occurred (Reyes et al. 2016). Its emergence has been recognized as a key innovation as it is seen to be homoplastic in extant angiosperms and is associated with species diversification (Woźniak and Sicard 2018). Indeed, radially symmetric lineages comprise fewer species than their bilaterally symmetric sister lineages (Sargent 2004; Woźniak and Sicard 2018). This suggests that zygomorphy confers advantages for flowers that facilitate more rapid diversification than their actinomorphic counterparts (Gómez et al. 2006).

Although there are many proposed hypotheses for the evolution of zygomorphy, they can be divided into three major groups from the perspective of benefits for the plant. The first one is that zygomorphy may increase flower (re)visitation by pollinators through making the flowers easier to perceive, learn or forage. Because zygomorphic flowers are morphologically more complex than non-zygomorphic ones, pollinators are likely to be given more visual information on which to base their specific recognition of these flowers (Neal et al. 1998). For example, zygomorphic flowers have a higher contour density, i.e., the dissected margin of flowers, compared to actinomorphic ones (Anderson 1977; Dafni and Kevan 1997). Lehrer et al. (1985) have shown that honeybees tend to scan the contour of flowers in a close range. This may suggest that zygomorphic flowers exploit scanning behaviour of certain pollinators and help them to reach the floral resources (Dafni and Kevan 1997). Additionally, a classical bee pollination syndrome, characterized by a common set of traits observed in various unrelated taxa pollinated by bees, includes tubular flowers. Such flowers are often zygomorphic and come in colours such as yellow, blue, or purple (Citerne et al. 2010). These bright colours could act as a guide to attract bee pollinators, along with a higher contour density. Alternatively, zygomorphic flowers often have elongated lower lips on which pollinators could land easily (Sprengel 1793). The resultant decrease in landing time may lead to increased return visits by experienced foragers (Neal et al. 1998). Thus, the complex shape of the corolla, coupled with its distinct colouration, may facilitate the recognition of the flower by specific pollinators, resulting in higher rates of revisitation.

The second group of hypotheses is that the complexity of the corolla restricts the type of pollinators that could exploit the floral resources. Floral complexity comprises various morphological aspects, such as zygomorphy, tube-like shapes and fused petals, which limit nectar access to only a small subset of animals (Neal et al. 1998; Zhao et al. 2016; Krishna and Keasar 2018). *Antirrhinum* and *Linaria* provide examples of such species, in which the lower lip presses tightly against the upper lip, to creating a physical barrier in front of the nectary. This selection for strong pollinators, which are capable of pushing their head between the two lips and opening the corolla (Citerne et al. 2010), resulting in morphological barriers that act as a filter, allowing only effective pollinators to access the nectar and contribute to its pollination (Krishna and Keasar 2018). In addition, the filtering of pollinators according to their ability to handle flowers is suggested to increase pollinator fidelity, as specialised pollinators mainly exploit fewer complex flower species rather than visiting many flower species with simple morphologies (Rodriguez-Girones and Santamaría 2007; Krishna and Keasar 2018). This fidelity of pollinators would benefit flowers in terms of lowering heterospecific pollen transfer (Krishna and Keasar 2018).

The third hypothesis suggests that a zygomorphic corolla restricts the movement of the insect (Wang et al. 2014; Ushimaru and Hyodo 2005; Neal et al. 1998), resulting in approaches that are more stable and predictable in direction (Fenster et al. 2009). As mentioned in previous paragraphs, zygomorphic flowers tend to conceal their nectar reward to select for pollinators that can handle the complex shape of the corolla (Citerne et al. 2010). The use of a lower lip as a guide has been observed by Sprengel (1793). This, combined with a horizontal orientation that forces the pollinator to approach from a consistent direction, would incentivise the pollinator to approach from the same angle to reduce handling time. Zygomorphy has been linked to a gene that suppresses the growth of the stamen (Rudall and Bateman 2004) and concentrates the reproductive parts of the flower in one location. Indeed, O’Meara et al. (2016) have shown that the diversification of zygomorphy is contingent on the presence of a corolla and reduction of the stamen. The concentration of the stamen and stigmas to a narrower area, combined with the predictable movement of the pollinator, would allow these reproductive parts to make more consistent contact with the pollinator’s body. This consistency would increase the conspecific pollen transfer while decreasing the heterospecific pollen exchange with pollinator-sharing species (Muchhala and Thomson 2010; Culbert and Forrest 2016). There have been other proposals for the evolution of zygomorphy, such as the protection of anthers and nectar from natural elements like rain (Sprengel 1793), or the “pollen placement hypothesis”, which proposes that the concentration of a plant’s reproductive parts in an area increases the likelihood of conspecific pollen transfer and reduces the risk of heterospecific pollen transfer.

Here we focus on the third group of hypotheses described, that zygomorphy restricts the angle of entry of pollinators. This effect has often been assumed in studies of floral evolution (e.g., Sargent 2004), but it has rarely been tested empirically. The effect of corolla shape on pollinator entry angle has been examined by Culbert and Forrest (2016) in a laboratory experiment using artificial flowers and bumble bees (*Bombus impatiens*). Using circular (radially symmetric) and rectangular (disymmetric) flowers, they showed that the approach consistency of bees was higher on disymmetric than on radial flowers. However, all the artificial flowers in their experiment were oriented horizontally, meaning that the flowers faced horizontally from the ground. While zygomorphic flowers are usually seen oriented horizontally in nature, radial flowers are commonly oriented in an upward or downward manner. Additionally, Fenster et al. (2009) have suggested that the entry angle stabilisation observed in zygomorphic flowers may be provided by the horizontal orientation in which they are presented, rather than by corolla symmetry. Therefore, the results of Culbert and Forrest (2016) may not fully represent the landing behaviour of pollinators, as they only present the artificial flowers in one orientation. To elucidate the relative importance of floral symmetry and orientation on the consistency of entry angle, it is necessary to compare flowers of different symmetry at different orientations.

In this study, we aimed to evaluate both the effect of floral symmetry and orientation on the consistency of pollinator entry angle. We used artificial flowers with three symmetry types (actinomorphy, zygomorphy and disymmetric) and oriented them in three ways (upward, horizontal and downward). By testing nine possible combinations of symmetry and orientation, we tried to dissect and quantify the effects of two floral features separately.

## Materials and methods

### Artificial flowers

**Fig. 1:**
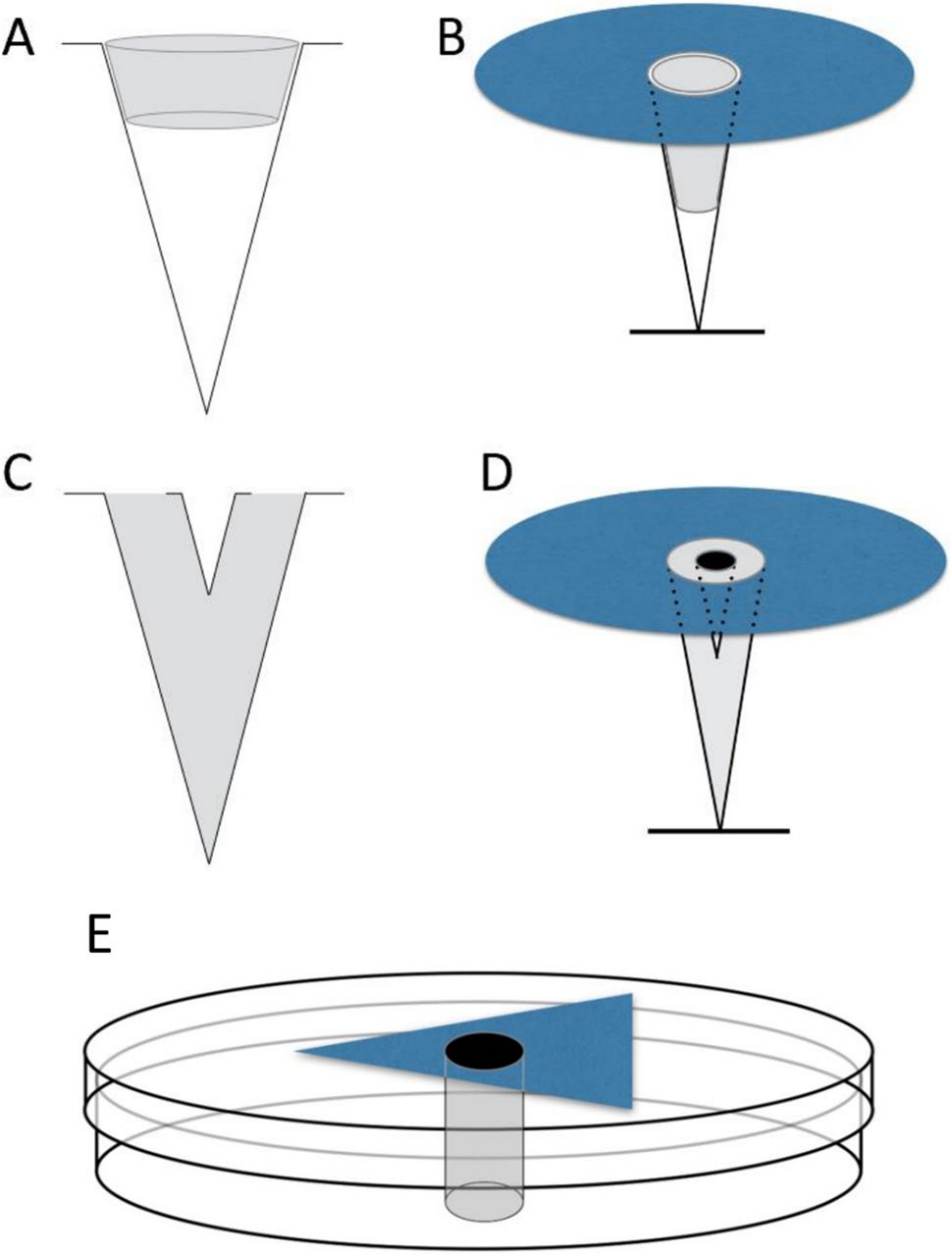
Diagrams of the artificial flowers used in this study. **A** The training flowers made from a 1.5-mL microcentrifuge and a small piece of dental cotton (shown in grey). **B** The artificial flower with the blue paper corolla attached. The diagram depicts a circular shape representing radial symmetry, although the same style applies to the other two shapes. Sugar solution was added directly into the microcentrifuge and the dental cotton was used to prevent the liquid from leaking out. **C, D** The test flowers, with C showing the configuration without the corolla and D showing it with the corolla. A 1.5-mL microcentrifuge was used as the base and filled with a white, odourless clay (shown in grey), in which a 0.5 ml microcentrifuge was added. A small piece of cotton was added into the 0.5-mL microcentrifuge where 30% w/w sucrose solution was added. **E** The petri dish (19 mm in diameter) flower. A triangular (bilaterally symmetrical) corolla is shown, but the configuration is the same for other corolla shapes. The same dental cotton used for the training flowers (**A, B**) was also used for this flower (shown in black). All types of flowers had the same corolla dimensions, and even though the dental cotton was larger than the microcentrifuge, the white clay in the test flowers made them look similar with the training flowers

We used artificial flowers for the experiments, each consisting of a “corolla” cut from blue drawing paper (~ 11.5 cm^2^), and a container for cotton, from which the sucrose solution (“nectar”) can be collected by bees. Each corolla shape represents one of the three types of floral symmetry: actinomorphy (circular; 38 mm [diameter]), zygomorphy (triangular; 40 mm [base] × 57.5 mm [height]), and disymmetry (rectangular; 55 mm [length] × 21 mm [width]). We used artificial flowers with identical appearance for the training and test phases, the only difference being the configuration of the cotton container (Fig. 1).

A test flower (Fig. 1A and B) had a 0.5-mL microcentrifuge tube as a container for a small piece of cotton. This microcentrifuge tube was embedded into a larger (1.5-mL) microcentrifuge tube using white, odourless clay. The opening of the smaller tube was at the same level as that of the larger tube. The cotton was inserted into the smaller tube, and the nectar was added on top of it so that bees could access it easily. The cotton was used as a fluid reservoir to prevent the nectar from leaking out of the microcentrifuge tube when the flower is not upwardly presented, while allowing bees to easily access it. On the other hand, a training flower (Fig. 1C and D) used a 1.5-mL microcentrifuge tube as the container, into which a dental cotton roll was inserted to prevent the nectar from leaking out. We chose a larger nectar reservoir for the training flower, so that the nectar would not run out quickly and the bees would not lose their motivation. We also used six plastic petri dishes (19 mm [diameter]) as supplementary feeders (Fig. 1E) during the training phase (two petri dishes per flower shape). Each petri dish had a hole cut into the middle and a dental cotton was inserted. It absorbed the nectar in the petri dishes and allowed bees to access the nectar. These were only used in the upwards orientation and acted as a larger reservoir of nectar to ensure that the bees would not run out of food.

**Fig. 2:**
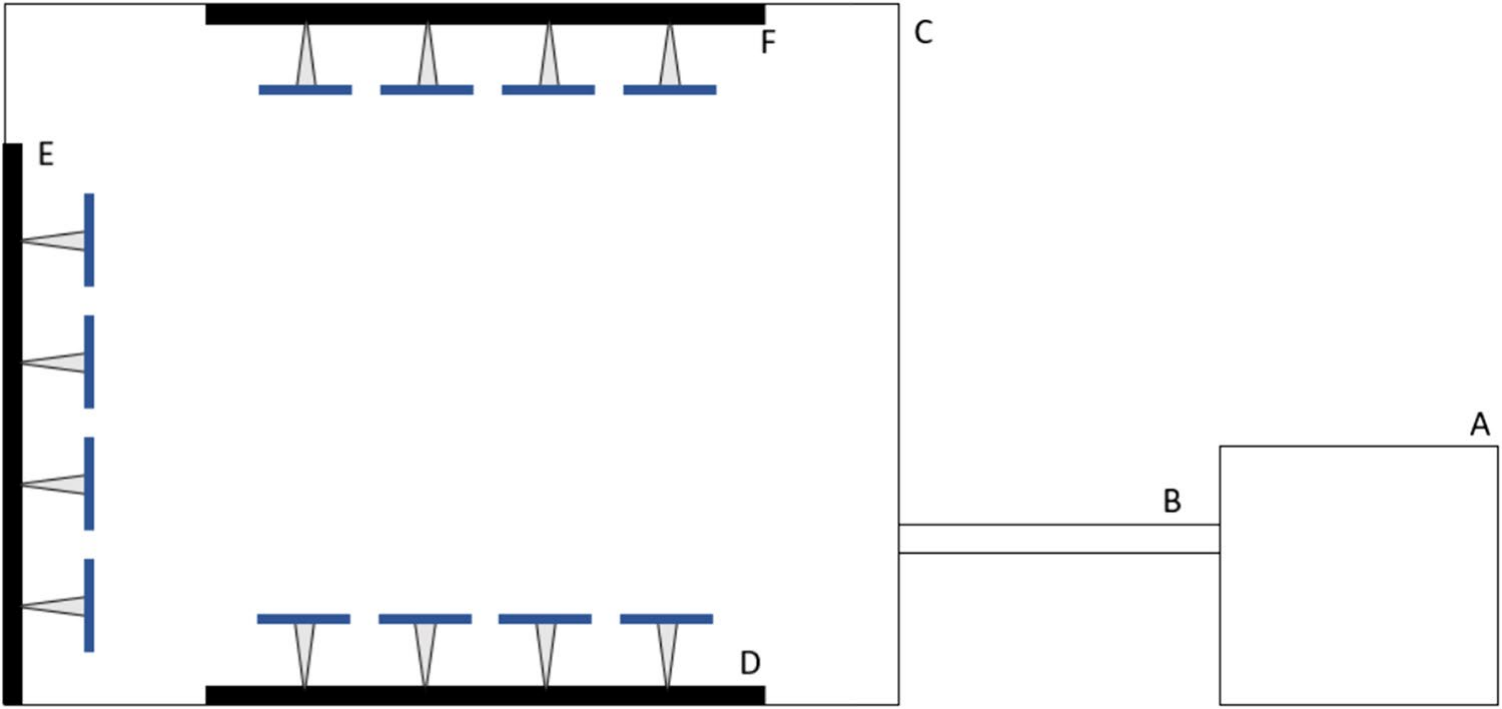
Diagram showing the configuration of the artificial flower grids. **A** colony; **B** tunnel connecting the colony to the flight cage; **C** flight cage; **D** flower grid for upwards orientation; **E** flower grid for horizontal orientation, **F** flower grid for downward orientation

Twelve flowers were positioned on a grid in a three-by-four pattern, with a 10-cm interval between the centres of the adjacent flowers. The grid was placed in one of the three positions (Fig. 2): floor (grid lying flat on the floor of the cage), wall (grid leaning against the back side of the cage at 90° to the ground), and ceiling (grid hung upside down from the ceiling with metal hooks). Hereafter, we refer to these positions as “upward”, “horizontal”, and “downward” respectively, to represent how each position determines the flower orientation. Within each grid, we aligned the corollas so that their symmetry axes all point in the same direction to minimize the possible effects of variable corolla alignment. For upward and downward presentations, both the longer symmetry axes of disymmetric flowers and the symmetry axes of zygomorphic flowers were aligned parallel to the line connecting the nest and the cage. Moreover, the sharpest vertices of zygomorphic flowers were directed towards the wall opposite to where bees enter the cage. For horizontal presentations, the zygomorphic and disymmetric flowers were aligned so that an approaching bee could view them as upright triangles or upright rectangles, respectively (Fig. 1D and E).

### Experimental procedures

**Fig. 3:**
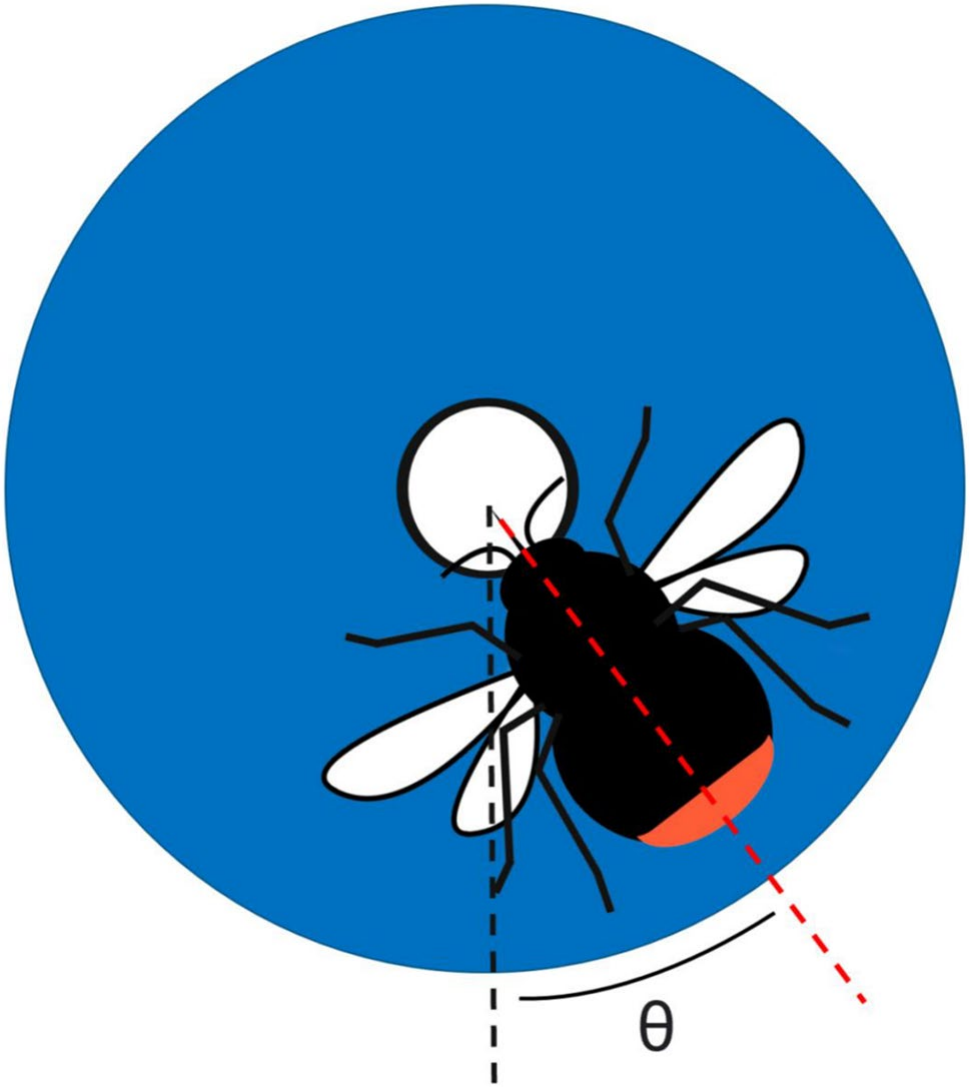
Schematic representation of the method used for measuring the entry angle of a bee. Two dotted lines were drawn from the centre of the artificial flower, one going down the middle of the flower (black) and another down the midline of the bee. We defined the black line as zero degree and measured the counter-clockwise angle between these lines on a 0–360° scale as the bee entry angle

We used workers from two commercial colonies of bumble bees, *Bombus ignitus* Smith, provided by Agrisect, Ibaraki, Japan. Colonies were maintained in nest boxes. The nest box (one at a time) was connected to a flight cage measuring 100 × 70 × 70 (H) cm through a transparent box equipped with gates, which allowed for the controlled entry and exit of individual bees into and out of the cage (Fig. 3). Pollen was supplied ad lib every day, directly into the colony.

During the training, which was performed before and between the test trials, we allowed the bees to forage freely in the cage by leaving the entrance open. Each training consisted of two phases: initial training phase and advanced training phase. During the initial training phase, a single training grid was placed on the floor of the cage (upward) (See Figure S1). In the grid, there were four flowers of each symmetry type arranged so that flowers of the same type were not next to each other. The initial phase was used to encourage bees to learn the association between the appearance of the corolla and nectar. Once bees started regular foraging on the training flowers, we proceeded to the advanced training phase, where two training grids were placed in the cage horizontally and downwardly, respectively (See Figure S2). The advanced training phase was necessary to allow the bees to familiarise themselves with the three orientations. We found that when using just the initial training phase, bees were reluctant to land on downwards facing flowers. We also added six petri dishes (two per corolla shape), which were haphazardly placed on the floor as supplementary feeders. The training flowers and petri dishes were filled with 20% (w/w) sucrose solution and were replenished appropriately. Once consistent foraging began, we uniquely marked reliable foragers on their thorax with numbered, coloured tags. The bees were exposed to all three corolla shapes and orientations until they finished the advanced training phase; based on the ID tags, we constantly checked whether individual bees gained sufficient experience with all the shapes and orientations.

Test trials were conducted using a single test grid placed in the cage. For each trial, we randomly selected one of the three symmetry types and arranged 12 of them in the grid, and then selected one of the three orientation types. In other words, one of the nine combinations of symmetry and orientation was haphazardly chosen for each trial. As a reward, 10-μL of 30% sucrose solution was used (the concentration was increased to boost the bees’ motivation for foraging). A marked bee was selected haphazardly to carry out the trial. Bee foraging was filmed with a video camera (GZ-MG575-S, JVC Kenwood, Yokohama, Japan) that was placed at 90° to the flowers. When the bee failed to land on a flower for longer than 5 min or attempted to return to the nest through the gated entrance, we considered the foraging trip to be over; these bees were allowed to return to the nest on their own, or were manually removed from the flight cage and returned to the nest. A total of 34 individuals were used (17 per colony). Each symmetry and angle combination had eight to 10 trials, for a total of 78 trials (See Table S1).

**Fig. 4:**
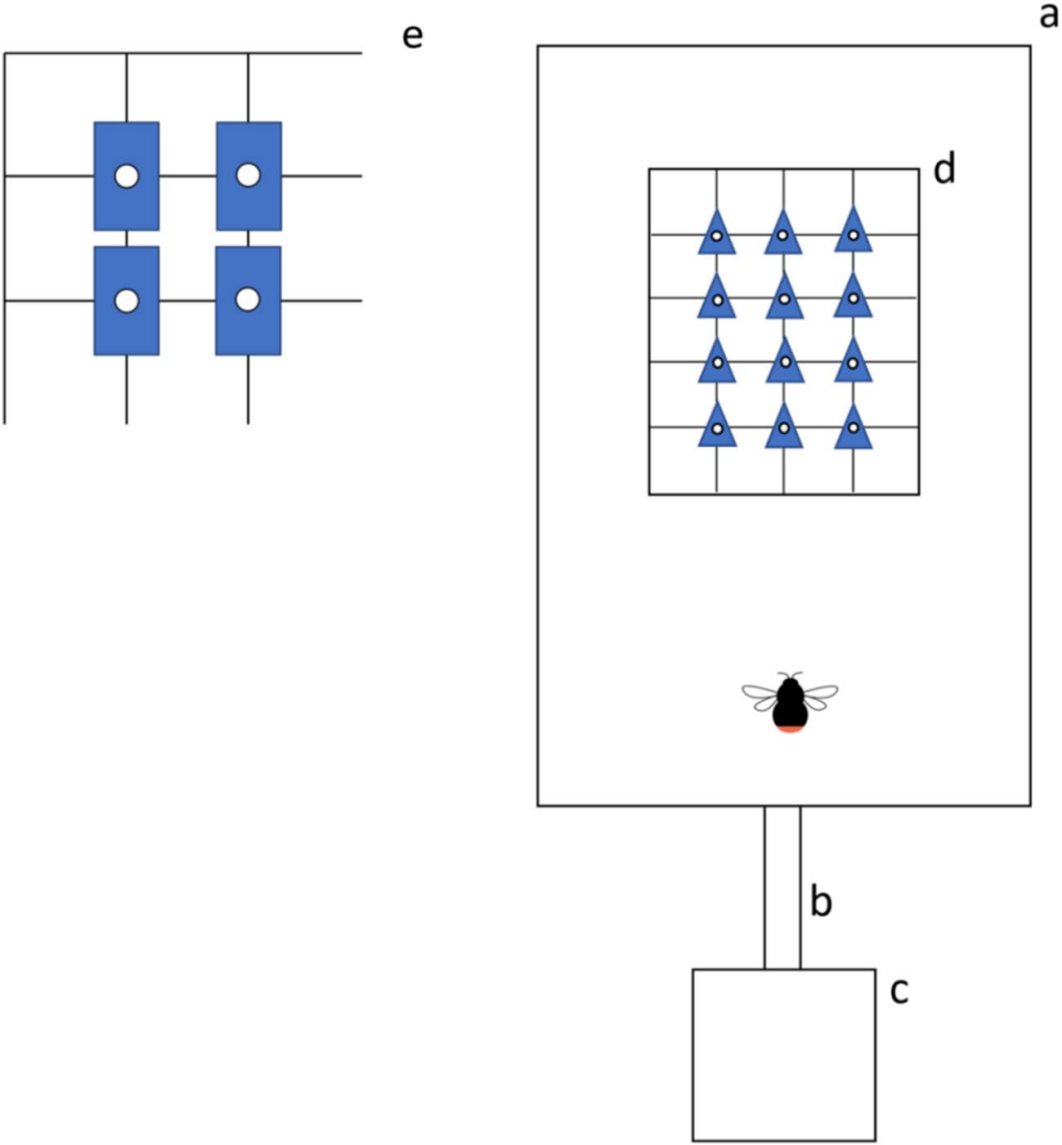
An aerial view of the experimental setup when flowers were oriented upwardly. **a** flight cage; **b** tunnel for bees to pass through with gated entrance; **c** colony; **d**, **e** grid of artificial flowers. The alignment is shown for zygomorphic **d** and disymmetric **e** flowers, respectively

After the experiment, we went through the videotaped images and took a screenshot of each successful landing. A successful landing was defined as the bee’s posture being stopped on a flower and her proboscis extended. For each of them, the entry angle was measured using ImageJ (National Institute of Health, Version 1.52q, 2019). We measured the angle between two lines extending from the centre of the flower, i.e., one which goes vertically down the middle of the flower and another goes down the midline of the bee’s body. We defined the vertical line as a zero degree and measured the counter-clockwise angle of the midline of the bee’s body on a 0–360° scales as the directionality of the bee’s entry (Fig. 4).

We also measured the time it took for the bees to land on each type of flower (hereafter, “landing time”) to check if floral symmetry or orientation affects the ease of landing on flowers for bees. This was conducted by placing a video camera at about 90°, 50–80 cm away from the flight cage, and recorded the sideview of the grid. We randomly selected three landings from each of the 4–6 trials per shape-angle combination, and counted the number of frames it took for the bee to land on the flower, using Windows Media Player (30 frames per second). Because a bee usually initiates a hovering phase, approximately 8 mm away from the flower, before moving forward to land (Reber et al. 2016), we started counting the frames when the bee first hovered in front of the target flower. The bee was considered to be hovering when it stayed in the same position for two or more frames. The counting was continued until the bee touched the flower with either of its legs and the legs remained on the flower during the successive two frames. This measurement was taken only for the second colony we used. A total of 16 individuals were used, with four to six trials per shape-angle combination, for a total of 38 trials (for more information, see Table S2).

### Statistical analysis

We first converted the data of entry angle from degrees to radians. We then calculated the circular variance (mean resultant length, MRL) for each trial, as the ratio of the observed length of the resultant vector to the maximum possible length of resultant vector for the same size of sample. The maximum possible length of resultant vector is obtained when all the entries were in the same direction. For actual computation of MRL, we used the *rho.circular* function in the R package “circular” (Pewsey et al. 2013). The MRL will take a value from one to zero, which is difficult to interpret in terms of actual angles. Therefore, we converted the MRL into circular standard deviation (circular SD) using the formula:

1 $$circular\;SD = \frac{180}{\pi } \bullet \sqrt{-2\bullet log \left(MRL\right)}$$

where π is the circular constant. The circular SD, like the usual standard deviation (SD), represents how much any one arbitrary data point deviates from the central value (mean entry angle) in unit of degrees (Pewsey et al. 2013).

Due to the shape of certain flowers, bee entry angles could follow non-unimodal distributions. For example, a bee may tend to land on a triangular (bilaterally symmetrical) flower so that the tip of its abdomen invariably points toward the base apex of the triangle. If the “tilted” entry occurs on both sides of the symmetry axis, the observed entry angle would follow a bimodal distribution. In such cases, the angle variance would be larger when two peaks are treated as separate angles than when they are viewed as mirror images of the same angle. For checking such possibilities, we conducted Hartigans’ dip test for unimodality. We also calculated the circular standard deviation using angles from 0 to 180°, instead of 0–360°, by subtracting any angles larger than 180 from 360 before converting the angles from degrees to radians.

We then fitted a generalised linear-mixed model (GLMM) with a logarithmic link and a Gaussian error distribution to the data to determine whether and how floral symmetry and orientation affected the variance of bee’s entry angle. The circular SD was used as the response variable. We considered floral symmetry and orientation as fixed effects, colony and bee individual as a random effect, together with an interaction term between symmetry and orientation. A type II Wald chi-square test was performed to determine the significance of the fixed effects and the interaction.

Because we measured the landing time as the number of frames in video image, we fitted a GLMM to this data, using a logarithmic link function and a Poisson error distribution. We calculated the average number of frames elapsed for a landing in each trial, and then used it as a response variable. Floral symmetry and orientations were considered fixed effects, bee individual as a random effect, together with the interaction term between symmetry and orientation. Colony was not added as a random effect as the individuals came from the same colony. A type II Wald chi-square test was performed to determine the significance of the fixed effects and interaction. For the graphical representation of the data of both the circular SD and landing time, the marginal (model-adjusted) means and standard error (SE) were estimated using the emmeans package in R (Lenth et al. 2019). To assess differences between means, post hoc tests were performed using pairwise contrast analysis. *P*-values were adjusted for multiple comparisons with false discovery rate (FDR) controlling procedures (Benjamini & Hochberg 1995).

## Results

**Fig. 5:**
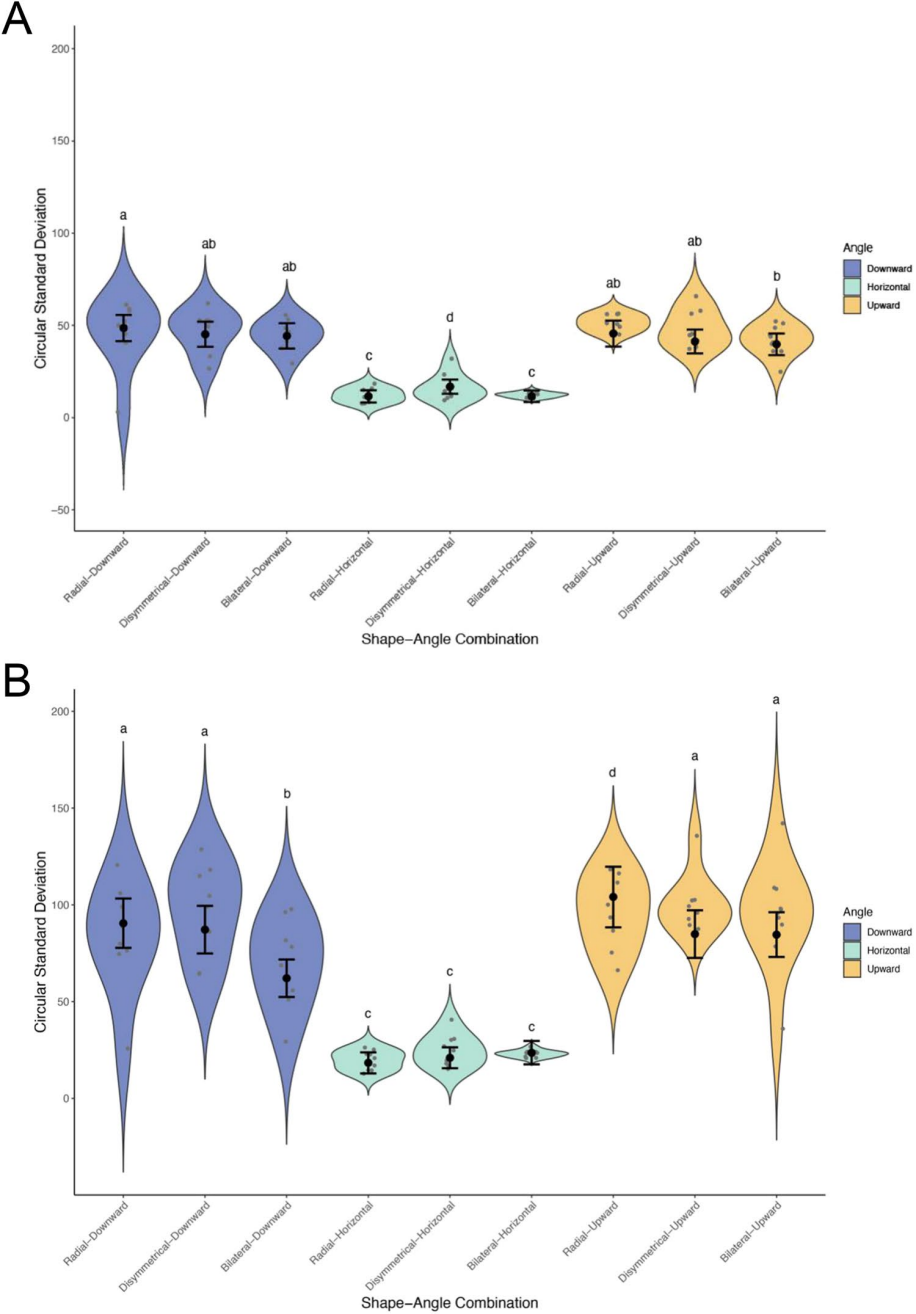
Circular variance of pollinator entry angle in flowers with different combinations of shape (symmetry) and angle (orientation). The y-axis and x-axis represent the circular standard deviation and the shape-angle combination, respectively. **A** Shows the results obtained with angles measured on a 0–360° scale, and **B** shows the results obtained with angles measured on a 0–180° scale, i.e., in clockwise and counter-clockwise directions. Error bars indicate the standard error of the circular SD for each combination. Means with shared letters indicate that there is no significant difference at a 0.05 alpha level. Significance levels are adjusted for multiple comparisons with false discovery rate (FDR) controlling procedures

We found that when using angles from 0 to 360°, all factors significantly impacted the variance of pollinator entry angle (Fig. 5A) (symmetry: χ^2^ = 20.06, *P* =  < 0.0001, type-II Wald chi-square test; orientation: χ^2^ = 340.67, *P* < 0.0001, type-II Wald chi-squared test; symmetry x orientation: χ^2^ = 21.52, *P* = 0.0003). On the other hand, when using angles ranging from 0–180°, only orientation significantly impacted pollinator entry angle (Fig. 5B) (χ^2^ = 241.92, *P* < 0.0001, type-II Wald chi-squared test), while symmetry and the interaction between symmetry and orientation showed no significant impact (symmetry: χ^2^ = 4.75, *P* = 0.093; symmetry x orientation: χ^2^ = 8.34, *P* = 0.08, type-II Wald chi-squared test).

**Fig. 6:**
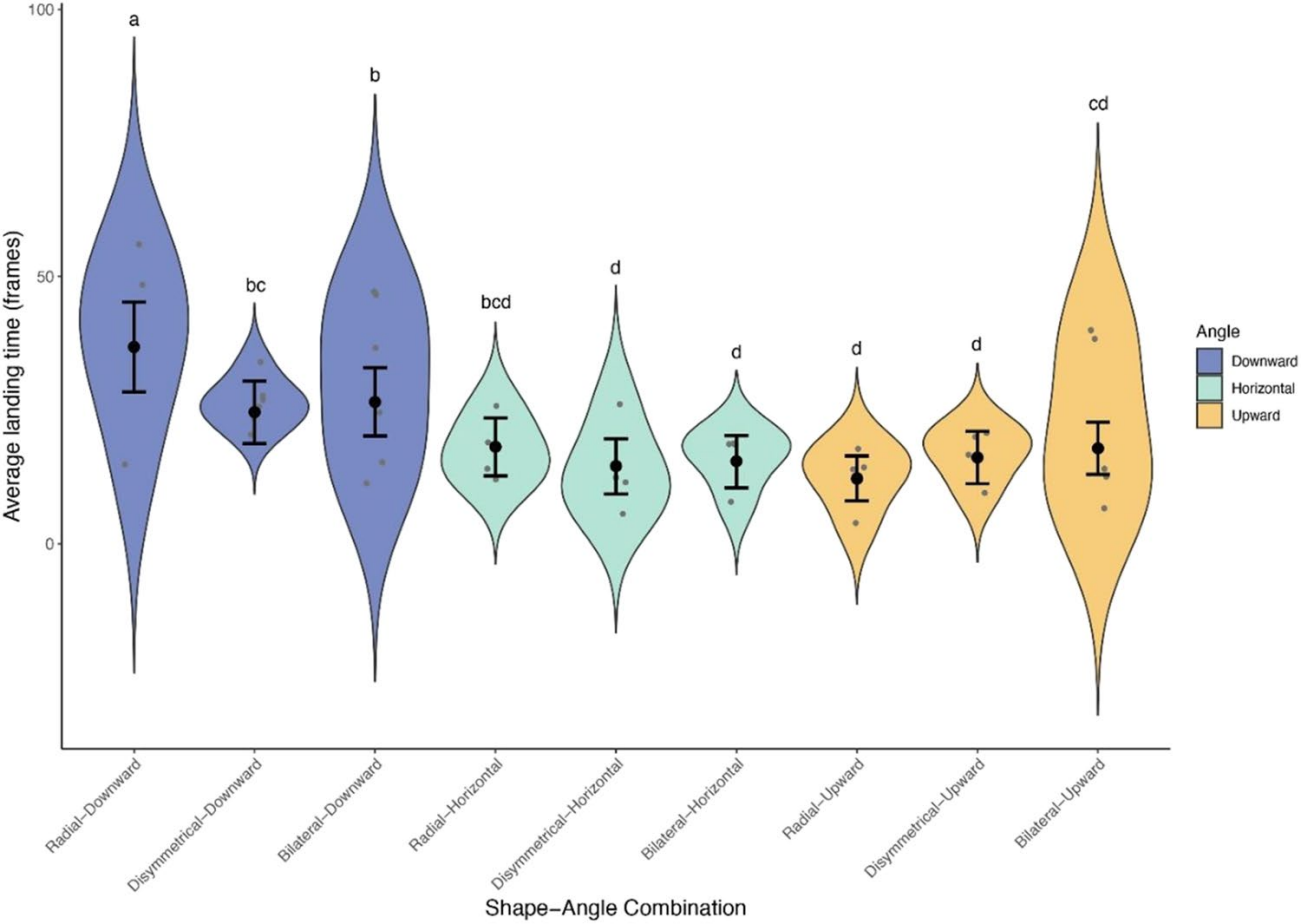
The average landing time (in number of frames) in flowers with different shape and angle, with the y-axis and the x-axis showing circular standard deviation and the shape-angle combination, respectively. Error bars indicate the standard error of the average landing time (frames) for each combination. Means with shared letters indicate that there is no significant difference at a 0.05 alpha level. Significance levels are adjusted for multiple comparisons with false discovery rate (FDR) controlling procedures

On the other hand, the landing time was significantly affected by orientation and the interaction between symmetry and orientation (Fig. 6, orientation: χ^2^ = 55.94, *P* < 0.0001, symmetry × orientation: χ^2^ = 13.11, *P* = 0.011, type-II Wald chi-square test), while it was hardly affected by symmetry (χ^2^ = 1.51, *P* = 0.47, type-II Wald chi-square test). The post hoc test suggests that bees took significantly longer landing on downwardly presented flowers than on upwardly or horizontally presented ones (Fig. 6).

## Discussion

As far as we know, this is the first experiment that examines both the effect of floral symmetry and orientation on the variance of pollinator entry angle. While our data showed a slight discrepancy between different angle ranges, it can clearly be seen that regardless of angle range used, that orientation had the largest impact on the variance of pollinator entry angle.

Since the publication of Sprengel (1793), it has often been assumed that zygomorphic corollas restrict the movement of a pollinator into the flower (e.g., Armbruster and Muchhala 2020). Based on this assumption, the pollen position hypothesis states that zygomorphy restricts the directionality of approach and movement of pollinators within and between flowers (Leppik 1972; Ostler and Harper 1978; Cronk and Moller 1997). However, our data shows that corolla symmetry is not the only contributor to pollinator angle consistency. We found that corolla symmetry affects entry angle consistency, but the effect is very small and becomes significant only when measured in 0–360° range (Fig. 5a). More importantly, the trend across the three types of symmetry is inconsistent across different orientations, as indicated by the significant interaction term. In contrast, our data show that floral orientation—specifically horizontal orientation—has the strongest stabilizing effect however we measured the entry angles (Fig. 5). Regardless of floral symmetry, bees approached flowers from more diverse directions when orientated upwardly or downwardly, resulting in increased variability of their entry angle compared to horizontally oriented flowers. Even when presented horizontally, zygomorphy and disymmetry did not increase the stability of the bee’s entry compared to actinomorphy (Fig. 5). Certain shapes in the methodology can lead to a bimodal distribution of pollinator entry angle. However, the results show that although not all trials exhibited unimodality (Table S3), this had a marginal impact on the results obtained (Fig. 5).

Our results are inconsistent with those of Culbert and Forrest (2016), who found that bumble bees entered disymmetric flowers at a higher consistency than they did for actinomorphic ones. Although we do not know the reason for this discrepancy, we could at least say that the stabilizing effect of disymmetric flowers found in Culbert and Forrest (2016) was much smaller than that of horizontal flowers in our study: the former found that standard deviations of entry angles were approximately 9° lower in disymmetric than in actinomorphic flowers; in contrast, we found that approximately 68° of difference in circular standard deviation of entry angles between horizontal and the other-oriented flowers (mean ± SE of circular SD of horizontal flowers = 22.5 ± 3.8°, degrees of freedom (df) = 58; upward flowers = 97.2 ± 3.9°, df = 67; downward flowers = 83.3 ± 4.1°, df = 67, estimated from the fitted GLMM). In other words, the stabilizing effect of floral orientation was more than seven times stronger than that of floral symmetry demonstrated in Culbert and Forrest (2016). It is also possible that our results, indicating that symmetry does not affect stability, were accentuated by the interspecific difference in bumble-bee body size. While the artificial flowers used in our study are similar in surface area to those of Culbert and Forrest (2016), *Bombus ignitus* have on average larger bodies than *B. impatiens*. This may have restricted the movement of the bees in our experiments as they had less space to land on the flowers.

The most probable reason why horizontal orientation had the strongest effect on the consistency of the bee entry angle (Fig. 5) is because bees typically fly with the ventral side of their body facing in the direction of gravity (downward). Fenster et al. (2009) also observed that hovering hummingbirds showed more consistent approaches when flowers were presented horizontally than when the orientation was vertical (upward) or semi-pendant. This was because the orientation of the flowers prevented pollinators from approaching the flower from the rear and sides. This, combined with the need for hummingbirds to stay upright while flying, forces a consistent approach angle (Fenster et al. 2009). While honeybees and bumble bees do not need to stay horizontal while flying like hummingbirds, the maximum angle of their body is limited (Evangelista et al. 2010; Reber et al. 2016), leading to a similar result where the pollinator is forced to approach the horizontal and downward-facing flowers from a consistent angle. These observations strongly suggest that the stabilisation effect does not come from visual guidance of the corolla shape or the existence of landing platforms on zygomorphic flowers, but the orientation of flowers relative to gravity. The forced directionality imposed by horizontal orientation, combined with the limited body angle of the bee to maintain stable hovering, would decrease the variation in landing angle. It has long been known that in nature, zygomorphic flowers are typically presented in a horizontal orientation (Ushimaru and Hyodo 2005). For example, in Nikkeshi et al. (2015), of the 36 flower species they used, 13 out of 15 bilateral species are horizontal (87%), while only two of the 21 radial species are horizontal (10%). In addition, Stewart et al. (2022) used six bat and 33 bee pollinated species. Of the bat pollinated species, two out of two bilateral flowers are presented horizontally (100%), while one of four radial species is horizontal (25%). Of the bee pollinated species, 11 of 12 bilateral species are horizontal (92%) and four out of 20 radial species are horizontal (20%) (Stewart et al. 2022). Therefore, much of our field impression that pollinators approach to zygomorphic flowers more consistently may have been heavily influenced or misled by this strong correlation between zygomorphy and horizontality.

Further, there is evidence that orientation is important for certain flower species. In one-sided racemes, changing the orientation of the flower resulted in fewer visit compared to the non-manipulated flower (Wang et al. 2014). This could indicate that in certain flowers, orientation is crucial to their recognition by pollinators. Changing the orientation of zygomorphic flowers could decrease the effectiveness of inflorescence attractiveness. However, bumble bees approached downward-facing flowers as much as horizontally facing ones, suggesting that pollinators that are specialised in handling downward-facing species can recognise opportunities for foraging despite changes to the orientation. In addition, there is evidence of self-righting capabilities in zygomorphic flowers (Armbruster and Muchhala 2020). This self-righting mechanism restores the “fit” of the pollinator to the flower, leading to higher levels of heterospecific pollen transfer, implying that orientation contributes strongly to the fitness of zygomorphic flowers.

Our data supports the idea proposed by Fenster et al. (2009) that the stability of pollinator entry angle would be first conferred by the evolution of horizontal flowers that have been driven by abiotic stress, such as rainfall. Fenster et al. (2009) also pointed out the possibility that horizontal orientation set the stage for the evolution of symmetry in sexual organs, as a result of its stabilising effect on pollinator entry. It is likely that horizontal presentation of flowers promoted the evolution of symmetric sexual organs in flowers through the increased accuracy of pollen placement. Studies have shown that both zygomorphy and horizontal/semi-pendant positioning increase the accuracy of pollen placement (Stewart et al. 2022), and that floral orientation and bilateral symmetry combined promote outcross-pollen transfer (Nevard and Vallejo-Marin 2022). While they showed little difference in the effect of zygomorphy and orientation, zygomorphic flowers are capable of reorienting themselves when they are damaged, whereas actinomorphic flowers are not (Armbruster and Muchhala 2020). This suggests that orientation is of particular importance for zygomorphic flowers. Considering that corolla symmetry had little effect on the stability of entry angle (Fig. 5), however, it seems questionable whether this horizontality eventually led to the evolution of zygomorphic corolla.

Our finding leaves open the question as to why zygomorphic flowers have evolved in the first place, especially in association with horizontal presentation. Given the current information, the most likely evolutionary advantage of zygomorphic corollas would be the restriction of pollinator type, to select for specialised and effective pollinators. This has been supported by empirical evidence (Lázaro and Totland 2014; Yoder et al. 2020). However, research has shown that changing the orientation of horizontally presented *Corydalis sheareri* reduces the number of visits by pollinators, indicating that a horizontal orientation is crucial for the detection of certain zygomorphic flower species (Wang et al. 2014).

Alternatively, zygomorphic flowers might increase the ease of landing through visual guidance, leading to an increased attractiveness for pollinators. This was unsupported by our landing time data (Fig. 6), in which no significant association was detected between corolla symmetry and landing time. The idea that zygomorphic corolla increases the attractiveness was also unsupported, at least in bumble bees (Culbert and Forrest 2016). There is a possibility that the evolution of zygomorphy may be driven by natural selection acting on the lower lips, serving as a landing platform for pollinators, and the zygomorphic corolla could be a developmental by-product of this selection. Future studies should explore the precedence of horizontality in the evolution of zygomorphy, as well as the selective advantages of a zygomorphic corolla or its associated traits, such as well-developed lower lips. The use of 3D flowers will make it possible to test whether the existence of lower lips on zygomorphic flowers, which are more common in nature than the 2D flowers we used here, could change the results on the effects of the association between zygomorphy and horizontal orientation on the consistency of pollinator entry angle. Finally, we also urge future studies to quantify the effect of floral symmetry and orientation on pollen transfer to see how plausible the previous assumptions are in terms of actual pollination accuracy.

In sum, we first attempted to dissect the entangled effects of corolla symmetry and orientation on the consistency of pollinator entry angle. We presented compelling evidence that the visual symmetry of corolla has only a small effect on the consistency of entry angle. Rather, we found that horizontal presentation of flowers plays the largest role in stabilizing pollinator entry. These results may force a reconsideration of the common conception about the evolutionary significance of zygomorphic flowers. That is, zygomorphic corollas have often been assumed to stabilize pollinator’s entry to flowers. This could be a misconception caused by the fact that zygomorphic flowers are typically presented at horizontal orientation. That is, horizontal orientation, rather than corolla symmetry, may have a greater impact on the stabilising of entry angle by forcing the pollinator to orient itself relative to gravity. We thus suggest that zygomorphy in and of itself may not restrict the entry angle of pollinators but may instead allow for the evolution of corolla shape differences which further restrict the entry angle, thus leading to more precise pollen placement. In this respect, our data are in line with Fenster et al.’s (2009) findings that orientation restricts pollinator landing behaviour, while inconsistent with Sprengel’s (1793) interpretation that floral symmetry causes a reduction in the variance of pollinator landing angle. Future studies are needed to determine how the stabilization effect of horizontal presentation affects pollination accuracy, as well as whether and how zygomorphic flowers have evolved and been maintained in many angiosperm lineages.

## Supplementary information

Below is the link to the electronic supplementary material.

Supplementary file1 (PDF 2606 KB)

## Data Availability

Data can be obtained from Nina Jirgal upon request.
