# Supplementary material for: Effects of floral symmetry and orientation on the consistency of pollinator entry angle
Source: Naturwissenschaften. 2023 May 16;110(3):19. doi: 10.1007/s00114-023-01845-w (PMC10185606; doi:10.1007/s00114-023-01845-w)

Effects of floral symmetry and orientation on the consistency of pollinator entry angle

Supplementary Information

Nina Jirgal[1,2*], Kazuharu Ohashi[2]

[1]University of Manchester, Oxford Road, Manchester M13 9PL, United Kingdom
[2]Graduate School of Life and Environmental Sciences, University of Tsukuba, Tsukuba, Ibaraki 305-8572, Japan

[*]Author for correspondence (Tel: +44 7588 361125, E-mail: ninajirgal@gmail.com)

Tables of contents

1. Supplementary tables
2. Pictures of training flowers and training layout
3. Pictures of experimental flowers

1. Supplementary tables

Table S1: Table showing all individual workers used in the entry angle experiment with the colony they belonged to. The "X" mark which combinations of flower shape and angle they took part in. On the left most column is the total number of trials each worker participated in. The bottom most column shows how many trials were conducted per shape and angle combination. The shape and angle abbreviations are as follows: R: radial (circular flowers); B: bilateral (triangular flowers); Di: dissymmetrical (rectangular flowers); H: horizontal orientation; Do: downward orientation; U: upward orientation.

		Shape-Angle combination									
BeeID	Colony	R-H	R-Do	R-U	B-H	B-Do	B-U	Di-H	Di-Do	Di-U	Trials per bee
22	1	X		X	X						3
20	1	X			XX						3
21	1	X	X			X	X	X			5
24	1		X								1
25	1			X							1
27	1									X	1
26	1	X					X	X			3
23	1							X	X	X	3
28	1				X						1
7	1	X		XX	XX			X			6
8	1									X	1
5	1						X				1
4	1		X					X			2
2	1						X		X		2
10	1									X	1
11	1						X				1
12	1					X					1
30	2	X	X	X							3
40	2						X				1
34	2					X					1
32	2					X		X			2
52	2								X		1
22	2					X					1
35	2		X	X	X		X				4
41	2	X	X			X	X		XX	X	7
51	2				X						1
55	2								X		1
21	2							XX		X	3
38	2	X			X			X			3
23	2			X						X	2
70	2		X						X		2
71	2					XX	X		X	X	5
88	2	X	X	X	X						4
93	2						X				1
Trials per combination		9	8	8	10	8	10	9	8	8	

Table S2: Table showing all individual workers used in the landing time experiment. The “X” mark which combinations of flower shape and angle they took part in. On the left most column is the total number of trials each worker participated in. The bottom most column shows how many trials were conducted per shape and angle combination. The shape and angle abbreviations are as follows: R: radial (circular flowers); B: bilateral (triangular flowers); Di: dissymmetrical (rectangular flowers); H: horizontal orientation; Do: downward orientation; U: upward orientation.

		Shape-Angle combination									
BeeID	Colony	R-H	R-Do	R-U	B-H	B-Do	B-U	Di-H	Di-Do	Di-U	Trials per bee
30	2	X	X	X							3
40	2						X				1
34	2						X				1
32	2					X		X			2
52	2								X		1
22	2					X					1
35	2		X	X	X		X				4
41	2	X	X			X	X		XX	X	7
51	2				X						1
55	2								X		1
21	2							XX		X	3
38	2	X			X			X			3
23	2			X						X	2
70	2		X						X		2
71	2					XX	X		X		4
88	2	X	X	X	X					X	5
93	2						X				1
Trials per combination		4	5	4	4	5	6	4	6	4	

Table S3: Table showing the percentage of trials per symmetry and angle combination which exhibited unimodality.

Treatment			
Symmetry	Angle	Total Trials	Unimodality
Radial	Horizontal	9	9 (100%)
Radial	Downward	8	8 (100%)
Radial	Upward	8	8 (100%)
Bilateral	Horizontal	10	9 (90%)
Bilateral	Downward	8	5 (62.5%)
Bilateral	Upward	10	6 (60%)
Disymmetrical	Horizontal	9	9 (100%)
Disymmetrical	Downward	8	8 (100%)
Disymmetrical	Upward	8	7 (87.5%)

2. Pictures of training flowers and training layout

Figure S1. Top: Birds eye view of training grid with petri dish supplementary feeders in the upwards orientation. This picture is taken during the first training phase where the association between the artificial flowers and a nectar reward is taught. Training grid feature all thee flowers shapes; circular (radial), triangular (bilateral), and rectangular (dissymmetrical). The supplementary feeders were added to increase the total amount of nectar available for the bees to forage; Bottom: Same setup as A but at a different angle.

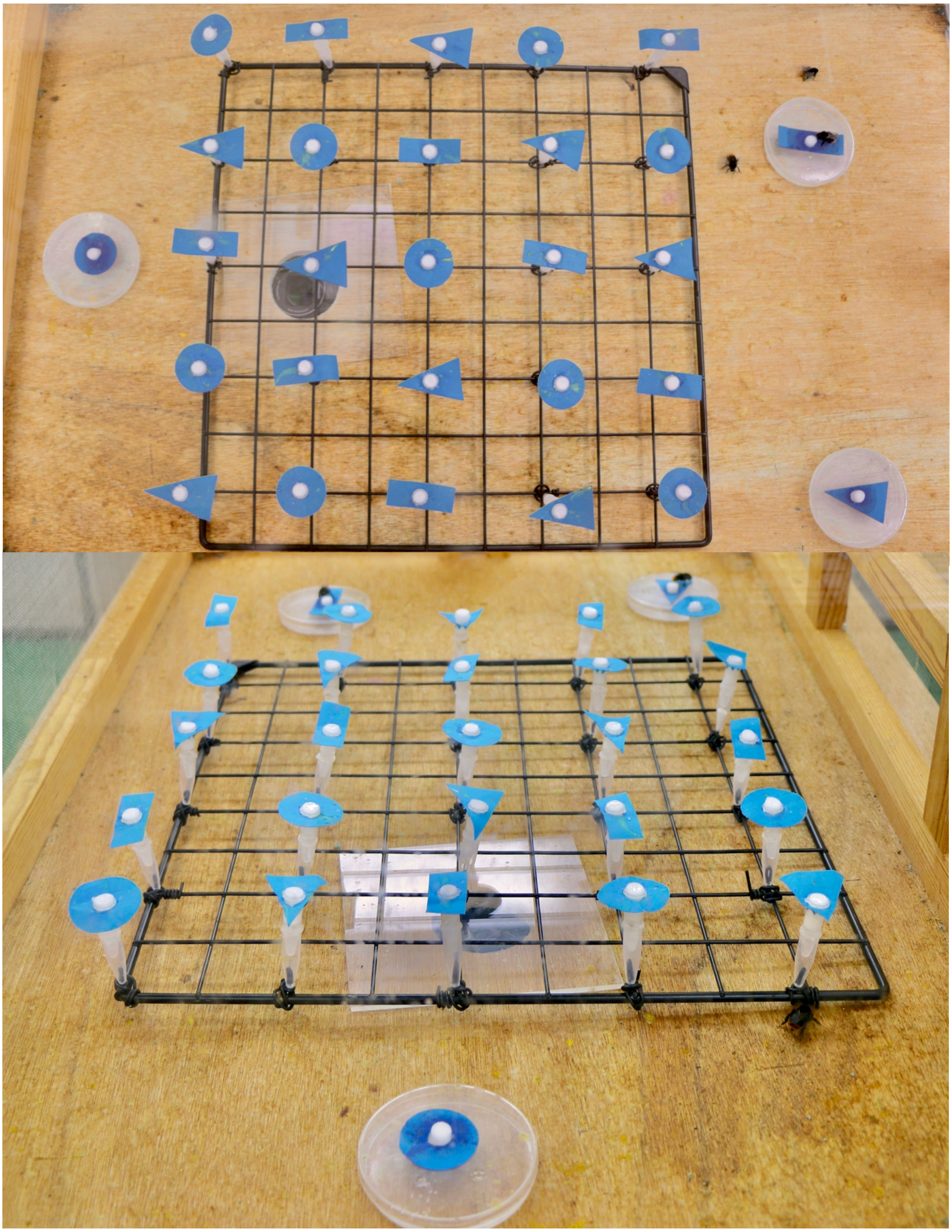

Figure S2: Left: Picture of advanced training phase. The grids are laid out in the horizontal position and downward position; B: Picture of advanced training phase with horizontal and upward position. The paper corolla on the supplementary petri dish feeders were replaced as the previous ones had become dirty.

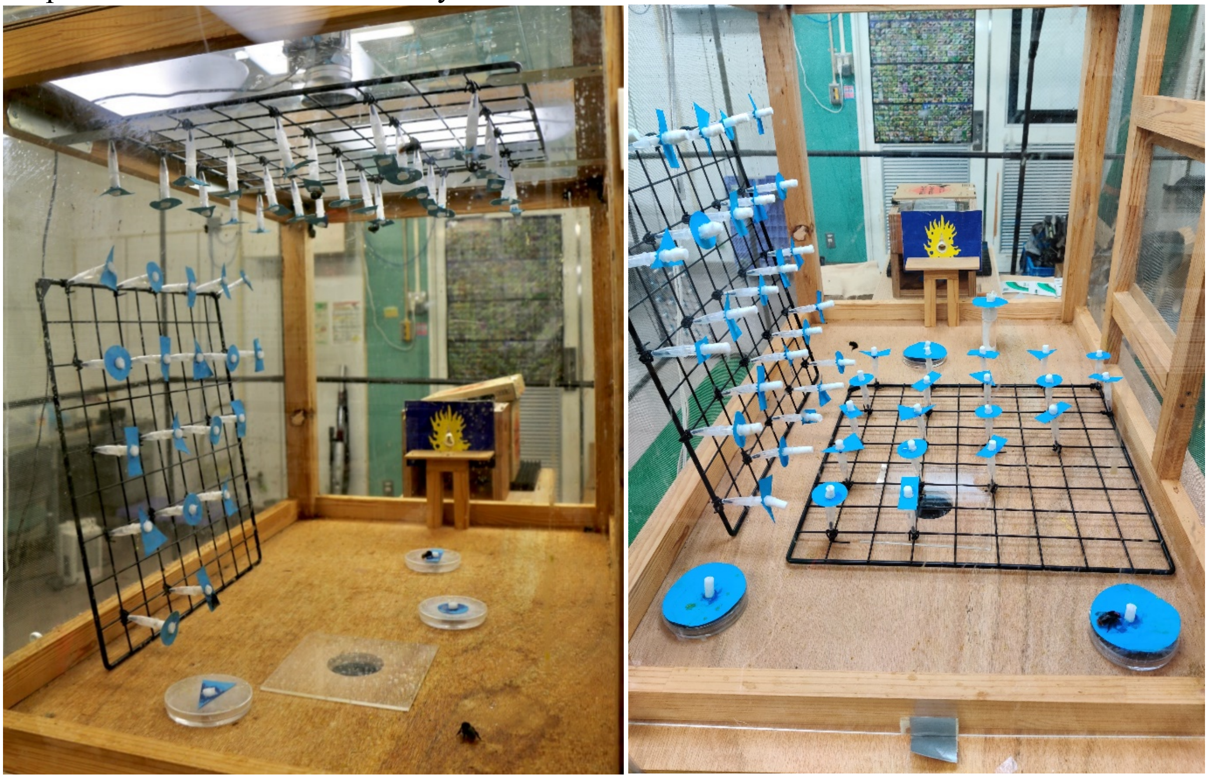

3. **Pictures of experimental flowers and experimental layout**

Figure S3: Left: Experimental grid with triangular (bilateral) flowers in the upward orientation; Top right: Experimental grid with triangular (bilateral) flowers in the downward orientation; Bottom right: Experimental grid with triangular (bilateral) flowers in the horizontal orientation.

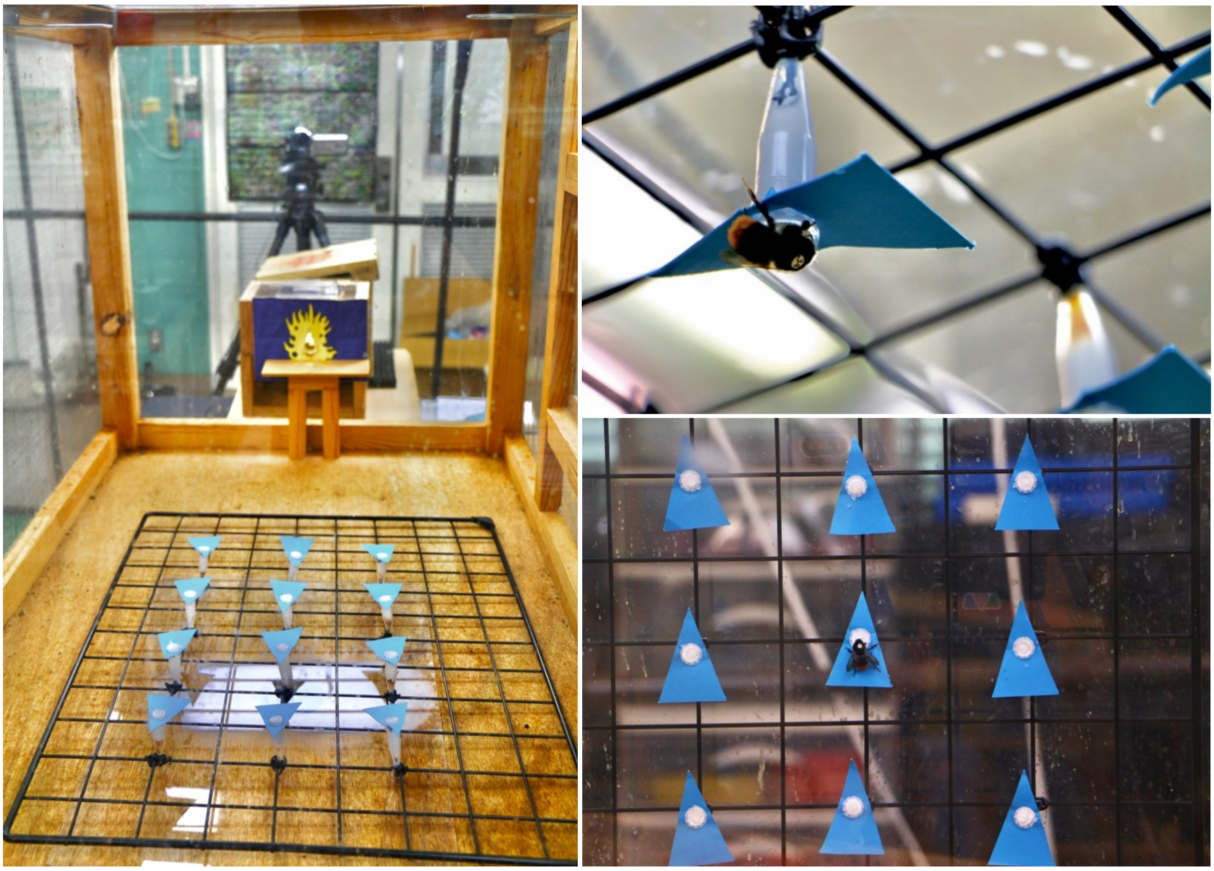

Figure S4: Left: Experimental grid with rectangular (disymmetrical) flowers in the upward orientation; Right: Experimental grid with rectangular (disymmetrical) flowers in the horizontal orientation.

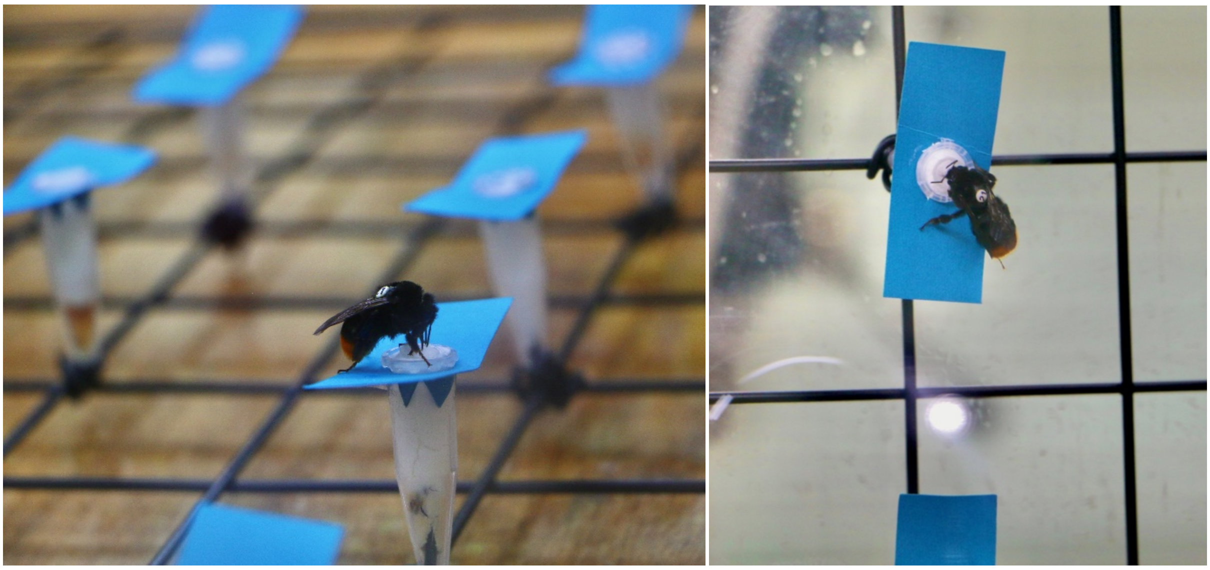

Supplement: Supplementary file 1 — Supplementary file1 (PDF 2606 KB) [file 114_2023_1845_MOESM1_ESM.pdf]
